# The INITIATE trial protocol: a randomized controlled trial testing the effectiveness of a “quit card” intervention on long-term abstinence among tobacco smokers presenting to the emergency department

**DOI:** 10.1186/s13063-021-05693-9

**Published:** 2021-10-23

**Authors:** Kerri A. Mullen, Aditi Garg, Frederick Gagnon, George Wells, Atul Kapur, Steven Hawken, Andrew L. Pipe, Kathryn Walker, Venkatesh Thiruganasambandamoorthy, Marta Klepaczek, Robert D. Reid

**Affiliations:** 1 Division of Cardiac Prevention and Rehabilitation, University of Ottawa Heart Institute, 40 Ruskin Street, Ottawa, Ontario K1Y 4W7 Canada; 2 Cardiovascular Research Methods Centre, University of Ottawa Heart Institute, 40 Ruskin Street, Ottawa, Ontario K1Y 4W7 Canada; 3 Faculty of Medicine, University of Ottawa, 75 Laurier Ave. E, Ottawa, Ontario K1N 6N5 Canada; 4 Ottawa Hospital Research Institute (OHRI), 501 Smyth Box 511, Ottawa, Ontario K1H 8L6 Canada; 5 Institute for Clinical Evaluative Sciences (ICES), Ottawa, Canada

**Keywords:** Nicotine replacement therapy, Smoking cessation, Tobacco dependence treatment, Health services research, Acute care, Emergency department, Behavioral counseling

## Abstract

**Background:**

Smoking cessation interventions implemented in emergency department (ED) settings have resulted in limited success, owing to factors such as lack of time, motivation, and incentives. A dynamic yet simple and effective approach that addresses the fast-paced nature of acute-care ED settings is needed. This study proposes a multi-center randomized controlled trial (RCT) to compare the effectiveness of an easy to deliver proactive, multi-component tobacco treatment intervention to usual care in the ED setting.

**Methods:**

This will be a prospective four-site, single-blind, blinded-endpoint (PROBE) RCT. Participants will be recruited directly in the ED and will be approached strictly in order of arrival time. Those randomized to the Quit Card Intervention (QCI) group will receive a “quit kit” which will include: a “Quit Card” worth $300 that can be used at any Canadian pharmacy to purchase any form of nicotine replacement therapy (NRT); a self-help booklet; and proactive enrolment in 6 months of telephone follow-up counseling. The usual care (UC) group will receive a “quit kit” which will include a brochure for a local smoking cessation program. Quit kits for both groups will be delivered in opaque, sealed envelopes, and identical in size and weight so to conceal group allocation from the blinded research coordinator. Randomization will be stratified by site and by the Canadian Triage Acuity Scale (CTAS), a value assigned to each ED patient based on the severity of the condition. An equal number of quit kits will be prepared for each CTAS level. The primary outcome will be bio-chemically verified smoking abstinence at 26 weeks. Secondary outcomes include smoking behavior at weeks 4, 52, and 104 as well as mortality and health care utilization outcomes. Investigators, outcome assessors, and data analysts will be blinded to group allocation until after primary analyses are completed. It is hypothesized that the QCI group will have higher a abstinence rate, improved health outcomes, and decreased healthcare utilization.

**Discussion:**

There are few examples of hospital EDs in Canada that systematically initiate tobacco cessation interventions for patients who smoke. Given the high smoking prevalence among ED patients and the relation of tobacco smoking to the majority of ambulatory care sensitive conditions, EDs are a missed opportunity in the initiation of tobacco treatment interventions. We have designed and will test an evidence-based tobacco treatment intervention that is simple and highly scalable.

**Trial registration:**

ClinicalTrials.govNCT04163081. Registered on November 14, 2019

## Administrative information

Note: the numbers in curly brackets in this protocol refer to SPIRIT checklist item numbers. The order of the items has been modified to group similar items (see http://www.equator-network.org/reporting-guidelines/spirit-2013-statement-defining-standard-protocol-items-for-clinical-trials/).

**Table:** 

Title {1}	The INITIATE trial: a randomized controlled trial testing the effectiveness of a “quit card” intervention on long-term abstinence among tobacco smokers presenting to the emergency department
Trial registration {2a and 2b}.	This trial is registered on ClinicalTrials.gov with the registration number NCT04163081.
Protocol version {3}	Issue Date: Jan 26, 2020 Protocol Amendment Number: 4 Author(s): KAM; AG; FG; GW; AK; SH; ALP; KW; VT; MK; RDR Revision chronology: • INITIATE Original – June 2019 • INITIATE Amendment 1 – October 29, 2019 o Minor changes: telephonic counseling schedule for Quit Card Intervention (QCI) participants • INITIATE Amendment 2 – February 3, 2020 o Minor change: Cellphone provided to study counselor to administer behavioral counseling to Quit Card Intervention (QCI) group • INITIATE Amendment 3 – February 24, 2020 o Minor change: make Baseline Case Report Form (CRF) a patient-facing document o Replaced SF-36 questionnaire with EQ-5D-QL survey tool to measure health-reported quality of life
Funding {4}	This trial is funded by the Canadian Institute of Health Research (CIHR), grant number PJT 162147
Author details {5a}	KAM^1*^, AG^1^, FG^1^, GW^2,3^ AK^3,4^, SH^4,5^, ALP^1,3^, KW^1^, VT^3,4^, MK^1^, RDR^1,3^ ^1^Division of Cardiac Prevention and Rehabilitation, University of Ottawa Heart Institute ^2^Cardiovascular Research Methods Centre (CRMC), University of Ottawa Heart Institute (UOHI), Canada ^3^Faculty of Medicine, University of Ottawa, Canada ^4^Ottawa Hospital Research Institute (OHRI), Canada ^5^Institute for Clinical Evaluative Sciences (ICES), Ottawa, Canada *Corresponding author at: Division of Cardiac Prevention and Rehabilitation, Ottawa Heart Institute Research Corporation (OHIRC), 40 Ruskin Street, Ottawa, ON K1Y 4W7, Canada. E-mail address: kmullen@ottawaheart.ca (K.A. Mullen).
Name and contact information for the trial sponsor {5b}	This trial is investigator-led. The OHIRC will ensure standard operating procedures and quality assurance regarding the trial. Contact of OHIRC: researchservices@ottawaheart.ca
Role of sponsor {5c}	This is an investigator-led trial. The trial funder (Canadian Institutes of Health Research) does not have any role in the study design, data collection, or management.

## Introduction

### Background and rationale {6a}

#### Background

In Canada, smoking results in nearly 40,000 premature deaths per year and it has been estimated that smoking-attributable healthcare use will cost approximately $80 billion over the next 20 years [1]. Heart disease and stroke are 2–4 times more common among smokers compared to non-smokers, and smoking accounts for 80–90% of all chronic obstructive pulmonary disease cases and 30% of all cancer deaths [2, 3]. Individuals who smoke have a 30–40% greater chance of developing type II diabetes, and those with diabetes have a greater risk of morbidity and premature mortality [4, 5]. Smokers use twice as many hospital days annually and are being hospitalized, on average, 12 years earlier than their non-smoking counterparts [6, 7].

Smoking cessation results in improvements in vascular, respiratory, and cancer-related health outcomes [3, 8], and reduces the prevalence and incidence of vascular events (e.g., acute myocardial infarction, stroke) substantially within the first few years [9, 10]. If a person quits before the age of 40, they will eliminate 90% of their risk of premature morbidity and mortality associated with smoking [8]. Hospital-initiated cessation interventions have been found to decrease 30-day, 1-year, and 2-year re-hospitalization rates, thus indicating the potential for improving “avoidable” hospital readmissions [6]. Despite progress in reducing the current smoking prevalence in Canada to 13%, there is a need for increased cessation efforts in order to reach Canada’s goal of 5% prevalence by 2035 [11]. Wider-scale implementation of proactive interventions initiated in clinical environments that treat large volumes of smokers is needed.

#### Rationale

Given the high smoking prevalence among patients treated in emergency departments (ED) [12], ED settings are an opportune and critical target area to initiate tobacco treatment and aftercare. Smoking rates among ED patients have been found to be >5% higher than levels in the general population [13, 14]. In a pilot study completed by our research team, an assessment of smoking prevalence completed at four hospital sites to be included in this study revealed that 509 of 2713 (19%) of patients reported current smoking. Therefore, the ED is an ideal environment to reach smokers and initiate tobacco treatment. However, few EDs in Canada offer tobacco treatment interventions to patients due to the environment’s fast-paced and acute care nature. Interventions in the ED must be simple and fast to deliver to facilitate implementation among ED staff.

Previous cessation intervention trials in ED settings have mostly found non-significant differences in abstinence rates (from −3.6 to 20%) between intervention and control groups [15]. Limitations of previous studies have included (1) low intensity of interventions (e.g., brief advice initiated in the ED and passive referral to a telephone “quit line”); (2) single-site studies, limiting generalizability; (3) weak-to-moderate methodological quality of most (88%); and (4) lack of health services outcomes (e.g., mortality, healthcare use, cost). Motivational interviewing (MI)-based counseling for smokers in the ED has shown to be effective; however, it has been acknowledged that, due to time constraints present in the ED, briefer interventions may be better suited for this setting [15]. A Canadian RCT compared advice from an ED physician and referral to a quit line to usual care (no support) [16]. In total, 660 patients were randomized to the intervention group, of whom 412 agreed to a quit line referral. Of those, only 13% enrolled in the quit line intervention, and only ~5% completed the program. Not surprisingly, given the low uptake, no significant differences in cessation were found between groups.

Clinical tobacco treatment interventions that include medication and behavioral counseling are most effective at helping people quit smoking [17]. NRT is the most frequently used cessation medication in acute care settings given its rapid effect on nicotine withdrawal. A systematic review and meta-analysis examining the effect of NRT reported an odds ratio (OR) of 1.84 (95% confidence interval (CI) 1.71–1.99)) for long-term cessation when comparing NRT to placebo. Combination NRT (e.g., pairing a nicotine patch with a shorter-acting form like gum, inhaler, or mouth spray) is more effective than nicotine monotherapy (OR 1.34, 95% CI 1.00–1.8) [18]. Behavioral counseling is crucial in supporting cessation efforts and preventing relapse. Proactive enrolment in behavioral support programs, as opposed to passive referral, is necessary. A recent study of a smoking cessation intervention in an out-patient surgery clinic found that 85% of patients enrolled in smoking cessation counseling when an “opt-out” referral approach was implemented [19]. This is compared to a 23% enrolment rate observed when hospitalized patients were referred to the same program using “opt-in” approaches [20]. A 2013 systematic review and meta-analysis of telephone counseling trials found positive effects for interventions involving multiple sessions of proactive counseling compared with self-help or single-session brief counseling (relative risk [RR] 1.41, 95% CI 1.20–1.66) [21].

Cost is a significant barrier to smoking cessation from the patient perspective. A study of 1400 hospitalized smokers in Ontario, Canada, by our research team found that 50% of participants belonged to the bottom two income quintiles [6]. Systematic review and meta-analysis data have shown full coverage of the cost of NRT, compared to no coverage, to increase quit attempts (OR 1.11, 95% CI 1.04–1.17; 4 trials) and abstinence (OR 1.77, 95% CI 1.37–2.28; 6 trials) in non-ED patient populations [22]. Even partial coverage has led to greater quitting compared to no coverage (OR 1.27, 95% CI 1.02–1.59; 5 trials). A systematic review examining the use of incentives (e.g., cash payments, gift cards) aimed at prompting or reinforcing smoking cessation among non-ED populations found that, compared to controls, smokers who received incentives were more likely to be abstinent for at least six months (OR 1.42, 95% CI 1.19–1.69; 17 trials) [23]. Unpublished pilot data from our group found that 43% of patients who initially reported they were not planning to quit smoking changed their status to ready after being offered coverage for their NRT in the form of a gift card, suggesting that incentives may be an important motivator among clinical populations (Mullen KA, Langevin E, Walker K, Noble S, Martin N, Reid RD: Nicotine replacement therapy “gift cards” as incentives to help smokers quit: a pilot study, unpublished). To date, the coverage of NRT or the use of incentives (e.g., gift cards) for smoking cessation has not been tested in the ED setting.

The evidence-based, proactive tobacco treatment intervention described in this protocol will be evaluated to inform future cessation interventions for smokers presenting to Canadian EDs. We propose a trial of high quality and generalizability that addresses many of the limitations and barriers faced by patients and staff of EDs. Our intervention (an NRT gift card called a “Quit Card” and proactive enrolment in behavioral support) has been designed to optimize uptake and smoking abstinence by including the most effective evidence-based behavioral and pharmacological approaches, removing specific challenges that smokers face when trying to quit (e.g., affordability, low confidence, and motivation), while packaging the intervention in a quick-to-initiate manner, making it ideal for fast-paced, complex ED environments.

### Objectives {7}

The main objectives of the INITIATE study are to determine whether, compared to usual care, smokers visiting an ED who receive a proactive tobacco treatment intervention initiated with a “Quit Card”:

- Will be more likely to quit smoking over the long term (≥6 months);
- Will have reduced rates of healthcare utilization and death (from 30 days to ≥ 2 years)?

### Trial design {8}

**Fig. 1:**
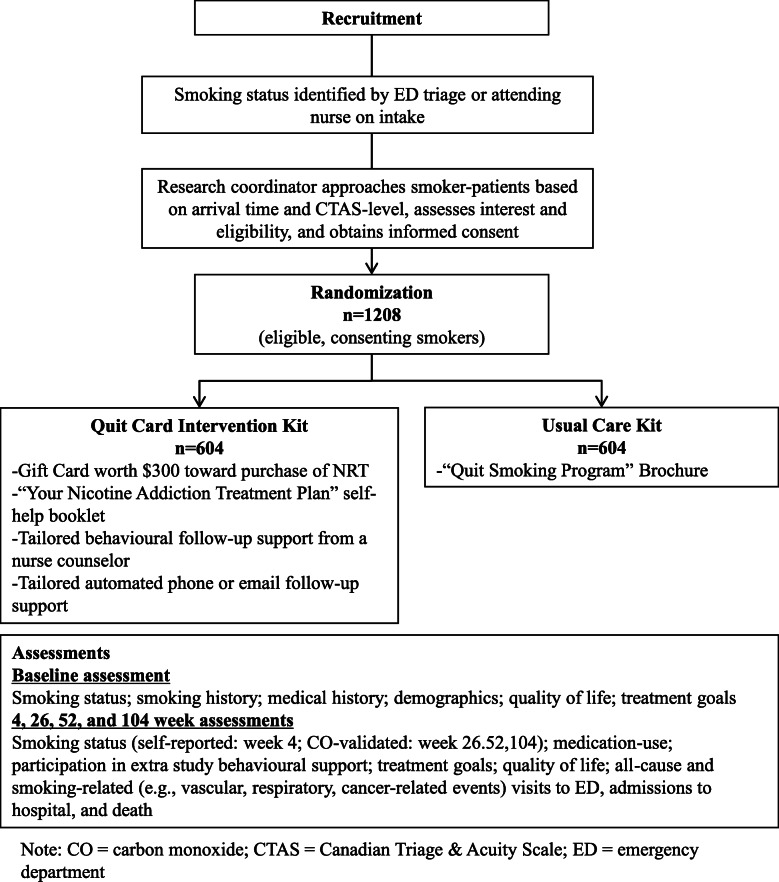
Flow diagram of INITIATE trial

This trial is a prospective, multi-site, randomized, single-blind, blinded-endpoint (PROBE) trial. Consenting participants will be randomized 1:1 to receiving usual care (UC, control group) versus the Quit Card-initiated intervention (QCI, treatment group). (See Fig. 1).

## Methods: participants, interventions, and outcomes

### Study setting {9}

We anticipate recruiting patients presenting to the ED at four hospitals which do not currently offer cessation support to ED patients and have >35,000 ED visits per year. The two primary recruitment sites will be two campuses of The Ottawa Hospital (TOH), an academic health sciences center located in Ottawa, Ontario. Additionally, participants will be recruited from Hôpital Montfort (urban) and Winchester District Memorial Hospital (rural community hospital).

### Eligibility criteria {10}

Participant inclusion criteria are the following:

1. Current daily smokers (smoke ≥ 5 cigarettes per day)
2. ≥ 18 years of age (the age of majority in Ontario)
3. Assigned a Canadian Triage and Acuity Scale (CTAS) level of 2–5 (emergent to non-urgent)
4. Able to read and understand English or French
5. Resides in Ontario and eligible for Ontario Health Insurance Plan (to permit linkage with administrative data housed at the Institute for Clinical Evaluative Sciences [ICES])
6. Available and willing to participate in follow-up assessments over the next 24 months
7. Has access to a telephone or computer
8. Able to provide informed consent.

Exclusion criteria are the following:

1. Currently participating in this or another smoking cessation study
2. Assigned a CTAS level of 1 (resuscitation—the most seriously ill patients with the highest likelihood of hospital admission) or in a psychiatric emergency unit
3. Pregnant, planning to become pregnant over the next 2 years, or breastfeeding
4. Has morbid illness which will prevent completion of 26-week follow-up (e.g., receiving palliative care)
5. In the ED physician’s opinion, manifests acute physical and/or psychiatric illness or has a cognitive impairment that would preclude participation in/benefit from the intervention.

### Who will take informed consent? {26a}

The research coordinator (RC) will be responsible for the recruitment of study participants and obtaining informed consent.

### Additional consent provisions for the collection and use of participant data and biological specimens {26b}

Additional consent will be obtained to link data to select healthcare administrative databases to evaluate health and health services outcomes. For consenting participants, study data will be individually linked to healthcare administrative databases at the Institute for Clinical Evaluative Sciences (IC/ES) using their unique Ontario Health Insurance Plan (OHIP) number.

### Interventions

#### Explanation for the choice of comparators {6b}

The quit card intervention (QCI) has been designed to optimize cessation outcomes. It has been shown that passive approaches (e.g., handing brochures) to tobacco treatment interventions for clinical populations result in lower rates of quitting (from 2.6 to 11.3%) [24, 25]. When medication and behavioral support are combined, long-term (≥6 months) abstinence rates approach 25–30% [26]. A “Quit Card” (STI Technologies, Halifax, Canada) to pay for NRT products was selected as (1) it reduces the barrier of cost for the participant; (2) it is a simple way to deliver intervention for the fast-paced ED; (3) hospitals do not have to worry about the storage and distribution of NRT product; (4) the program is only charged for the actual amount of product purchased by the patient; therefore, any unused value on the card is not wasted and can be applied to help more patients; (5) the card is programmed to pay for only NRT-specific Natural Health Product Numbers; (6) pharmacies process the card the same way they would any other health benefits insurance card; and (7) card-use, including date, location, NRT type, dose, and cost are tracked in real time.

A recent evaluation of a Quit Card pilot program for hospitalized smokers in Ontario, Canada, reported that out of 5722 individuals who received a $450 Quit Card, 70.2% used it and when used, >90% of its value was spent (Mullen KA, Langevin E, Walker K, Noble S, Martin N, Reid RD: Nicotine replacement therapy “gift cards” as incentives to help smokers quit: a pilot study, unpublished). The self-reported abstinence rate at 1 month was significantly higher among the subsample of participants that received the Quit Card (*n*=143) compared to a pre-incentive control (*n*=128) that did not (48% vs. 29%; OR 5.6 [2.5–12.5]; *χ*^2^= 30.6). Even among participants who indicated that they were “not ready” to quit, >50% redeemed their card for NRT and close to 30% reported abstinence at 1 month. Of those who received a Quit Card, 76% engaged in behavioral support, compared to 31% of controls (*χ*^2^=53.6; *p* <0.001).

### Intervention description {11a}

#### Usual care (UC) group

UC will be standardized across participating EDs. Eligible consenting participants randomized to the UC group will receive a kit that contains a brochure for the University of Ottawa Heart Institute’s Quit Smoking Program (QSP), a 6-month nurse-led behavioral counseling program. The brochure will include a description of the QSP program and information about how to register. UC participants will be able to self-register in the program if they so choose.

#### “Quit Card” intervention (QCI) group

Eligible consenting participants randomized to the QCI group will receive a kit that contains a “Quit Card” worth $300 that they can take to any pharmacy to purchase any type and brand of NRT (an approximate 6- to 12-week supply of combination NRT), and they will also receive proactive, tailored behavioral support from a study counselor for 26 weeks. QCI participants will have up to 2 months from study entry to use the full value of their Quit Card to encourage quit attempts. If a participant’s Quit Card expires before they use the full value and they would like to continue NRT, it will be possible for the study counselor to extend the expiration date. The study counselor will call QCI participants within the first week of study entry to initiate the behavioral support. Participants will receive at least four additional proactive (live) counseling calls, plus three automated calls or emails (based on participant preference) over 6 months (Table 1). During the counseling calls, the counselor will use standard follow-up protocols based on established baseline treatment goals [17]. The automated messages will be motivational and will include tips and reminders of what number to call should a participant wish to speak with the counselor for support [27].

**Table 1:** Behavioral support schedule

	Days after study enrolment						
	Days 3–7	Day 14	Day 45	Day 60	Day 90	Day 120	Day 150
Live call	X	X	X		X		
Automated call or email				X		X	X

#### Criteria for discontinuing or modifying allocated interventions {11b}

If a participant chooses to withdraw from the intervention, the intervention will stop at the moment of the participant’s request. If a participant consents to the use of their data, all data obtained up to that point of the request will be retained and utilized for subsequent data analysis in the duration of the trial.

The proposed intervention of the use of NRT for participants allocated to the QCI group is considered low risk. A systematic review and meta-analysis (92 RCTs, 28 observational trials) reported NRT patch-use was associated with increased skin irritation (OR 2.80, 95% CI, 2.28–3.24) and oral NRTs associated with mouth and throat soreness (OR 1.87, 95% CI, 1.36–2.57), mouth ulcers (OR 1.49, 95% CI, 1.05–2.20), hiccups (OR 7.68, 95% CI, 4.59–12.85), and coughing (OR 2.89, 95% CI, 1.92–4.33) [28]. A network meta-analysis (21 RCTs) found no evidence of increased risk of serious cardiovascular events with NRT (RR 1.95, 95% CI 0.26–4.30); however, it did report an elevated risk, mostly due to less serious events such as heart palpitations (RR 2.29, 95% CI 1.39–3.82) [29]. These reported risks are irrefutably much lower than those associated with continued smoking. If prolonged side effects are seen, NRT products used by participants allocated to the QCI treatment group may be modified.

#### Strategies to improve adherence to interventions {11c}

The behavioral counseling schedule has been designed to contact patients at important time points in terms of relapse prevention and Quit Card redemption reminders.

#### Relevant concomitant care permitted or prohibited during the trial {11d}

The use of other smoking cessation aids (e.g., varenicline, bupropion, vape devices) or participation in other smoking cessation interventions or programs will be queried during follow-up assessments and controlled for.

#### Provisions for post-trial care {30}

Upon request, study participants from either allocated group may be eligible for smoking cessation support post-trial.

#### Outcomes {12}

##### Primary outcome

The primary outcome will be smoking abstinence at 26 weeks, biochemically verified using an expired CO test.

##### Secondary outcome

Secondary outcomes related to smoking behavior include the following:

- Prolonged (since last follow-up assessment) abstinence, at 4, 26, 52, and 104 weeks
- Point prevalence abstinence at 4, 52, and 104 weeks
- Smoking reduction (i.e., change in the number of daily cigarettes) at 4, 26, 52, and 104 weeks
- Number of quit attempts since study entry at 4, 26, 52, and 104 weeks
- Use of cessation medication and/or e-cigarettes at 4, 26, 52, and 104 weeks
- Use of behavioral supports (e.g., counseling, quit lines) at 4, 26, 52, and 104 weeks
- Health-related quality of life (HR-QoL) at 52 and 104 weeks

Secondary outcomes related to health services include the following:

- All-cause and smoking-related (e.g., vascular, respiratory, cancer-related) visits to ED, admissions to hospital, and mortality at 4, 26, 52, and 104 weeks
- Cost-effectiveness ratios at 52 and 104 weeks

#### Participant timeline {13}

Please see Fig. 2.

**Fig. 2:**
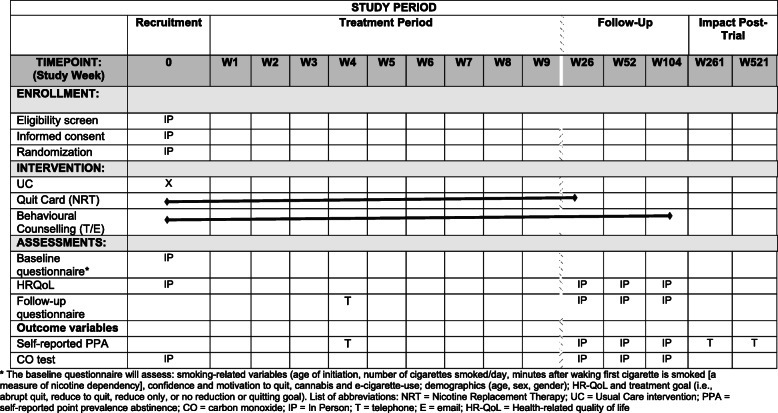
Schedule of recruitment, interventions, and assessments

#### Sample size {14}

The primary outcome of smoking abstinence at 26 weeks was used to calculate the sample size (PASS 16.0.2, www.ncss.com). Using logistic regression, with 905 participants assigned in a 1:1 allocation to the two groups, we will have 80% power to detect a clinically important 5% difference in smoking abstinence rates between the QCI and UC groups and >90% power to detect a 10% difference (two-sided test; alpha = 0.05). While we have included features to minimize attrition, we have increased our sample to account for a 25% loss to follow-up [30]; therefore, we intend to recruit 1208 participants to allow for per-protocol analyses.

In our assessment of intervention effect on health services, using a two-sided log-rank test and accounting for the competing risk of death, with 1208 participants we will have >90% power to detect a hazard ratio of 0.63 for all-cause re-hospitalization and 77% power to detect a hazard ratio of 0.82 for visits to the ED over 2 years (25% loss to follow-up; alpha = 0.05). We will have >90% power to detect a hazard ratio of 0.45 for death. This assumes cumulative incidence proportions for hospitalization of 0.45 for the UC group and 0.34 for the QCI group, ED visits of 0.68 for UC and 0.66 for QCI, and death of 0.15 for UC and 0.07 for QCI, based on a trial of hospitalized smokers conducted by our group [6].

#### Recruitment {15}

On average, 70,000 patients visit each participating ED annually, of which approximately 13,000 are current smokers. Each participating hospital will see 20–25 potential participants during “daytime” shifts (7am–7pm) or two per hour. Based on unpublished pilot data gathered from the TOH Civic campus ED in April 2017, 58% of smoking participants who were approached with a “Quit Card” in the waiting area accepted the intervention. Therefore, we estimate that our RC will spend 5 h per day, 5 days per week recruiting participants into both treatment arms of the trial. With approximately 9 smokers every 5 h, we expect that on average 5 smokers per day will be eligible and will consent to participate. Recruitment is expected to take 14 months.

### Assignment of interventions: allocation

#### Sequence generation {16a}

Group allocation will be determined using a random sequence computer generated by a statistical consultant (from the University of Ottawa Heart Institute’s Cardiovascular Research Methods Centre). Randomization will be stratified by two factors: participating site and CTAS level at intake. Two CTAS levels will be used for stratification, the first combining CTAS levels 2 (emergent) and 3 (urgent) and the second combining CTAS levels 4 (less urgent) and 5 (non-urgent).

#### Concealment mechanism {16b}

Prior to recruitment, an equal number of QCI and UC kits will be prepared in opaque packaging, identical in appearance and weight so that the allocation of the next participant will be unpredictable. Kits will be arranged sequentially and kept in two boxes, labeled based on CTAS level.

#### Implementation {16c}

The RC will be responsible for participant enrolment. The RC will approach potential participants strictly based on arrival time to the ED, will assess interest and eligibility, and obtain informed consent. For each new participant, the RC will select the next available kit in the corresponding CTAS level box. Participants will be asked to wait until after the RC has left to open their kits in order to maintain blinding. For those in the QCI group, their Quit Card will need to be registered upon study entry. To maintain RC blinding, each kit (both UC and QCI) will have a sticker on the outside envelope printed with a unique and random card registration number. The RC will enter this number into the Quit Card online portal. Numbers corresponding to true Quit Cards will register the cards, allowing the patient to purchase their NRT. Numbers corresponding to UC kits will be “dummy” codes that when entered into the portal will lead to no further action. Since the RC will not be able to explain the contents of the kits to study participants, both QCI and UC kits will contain simple instructions and contact information for the unblinded research manager if additional questions arise.

### Assignment of interventions: blinding

#### Who will be blinded {17a}

The principal and co-investigators, RC, outcome assessors, and data analysts will be blinded to group assignment for the duration of the study. Data tables incorporating coded group allocations will be presented at specific trial stages to the investigators. Both treatment arms will be grouped according to a specified code, which will not be known to the individuals. Study participants will also be blinded to specific intervention components to reduce performance bias.

#### Procedure for unblinding if needed {17b}

The research manager will receive a password-protected log with each kit number and allocation from the statistical consultant who completed the randomization. In the event of an emergency and/or a situation where the safety, care, and best interest of the participant is compromised by not knowing the treatment allocation, the research manager will initiate unblinding procedures.

### Data collection and management

#### Plans for assessment and collection of outcomes {18a}

The RC will collect baseline data in the ED during recruitment. Blinded research assistants will assess outcomes at week 4 by telephone and weeks 26, 52, and 104 in person. Smoking status will be assessed initially by a self-report of point prevalence abstinence (“Have you used any form of tobacco, even a puff, in the past 7 days?”) and confirmed via expired CO concentration testing using a CO meter. CO levels below 4 ppm will be considered confirmatory of non-smoking [31].

Secondary outcomes related to smoking behavior will be collected by survey and will be gathered in accordance with data-standard guidelines developed by the Ontario Tobacco Research Unit [32]: “How many cigarettes do you smoke per day on the days that you smoke?”; “In the past number of weeks, how many times did you stop smoking for over 24 hours because you were trying to quit?”. To evaluate health services outcomes, participant data will be individually linked to healthcare administrative databases at IC/ES using a unique Ontario Health Insurance Plan number. Mortality data will be acquired through the Registered Persons Database, ED-use data through the Canadian Institutes of Health Information (CIHI)’s National Ambulatory Care Reporting System, and hospital admissions through CIHI’s Discharge Abstract Database. To assess cost-effectiveness, costs associated with the delivery of both the QCI and UC conditions will be gathered. Cost per quality-adjusted life-year gained will be calculated using HR-QoL, collected with the EuroQol 5-dimension 5-level survey (EQ-5D-5L).

Satisfaction and perceived program barriers and facilitators will be assessed for both patients (at 26 weeks) and providers (at the end of each ED’s recruitment period) by open-ended survey questions asked to participants during the 26-week survey.

#### Plans to promote participant retention and complete follow-up {18b}

Overall, we expect high compliance for this smoking cessation trial. Quit cards are likely to be redeemed for medication. Pilot data found that 71% of patients who received a Quit Card used NRT within the first month. Moreover, we expect that participation in behavioral support programs will be followed through to completion. Data from an RCT of the effectiveness of the Ottawa Model for Smoking Cessation (OMSC) program’s behavioral support program observed call completion rates of 83% at day 3, 87% at day 30, and 87% at day 60 [27].

Participants will be compensated for the completion of each assessment in the trial, including baseline and follow-up questionnaires and CO tests. A $25 gift card will be provided to each participant completing the baseline questionnaire, a $10 gift card for the week 4 telephone survey, and $25 gift cards at each subsequent follow-up (total possible $110). In addition, costs associated with follow-up visits will be reimbursed (e.g., parking, transportation costs).

#### Data management {19}

Data will be managed using REDCap electronic data capture platform, where data are entered into a secure, web-based database that complies with Good Clinical Practices and regulatory requirements. The software includes an audit trail, participant and data entry status tracking, real-time data validation checks, and data extraction functionalities. The primary research team (PI, manager, RC, and assistants) will meet weekly to identify and mitigate any potential problems.

#### Confidentiality {27}

All personal health information identifying potential and enrolled participants collected during the study will be stored in paper files, kept in a locked filing cabinet in a restricted access area of the UOHI, or in encrypted electronic files on encrypted (password-protected; only study staff and the principal investigator will know the password) laptops. Details personally identifying participants, e.g., name, address, email) will not be used in data analyses.

#### Plans for collection, laboratory evaluation, and storage of biological specimens for genetic or molecular analysis in this trial/future use {33}

N/A

### Statistical methods

#### Statistical methods for primary and secondary outcomes {20a}

For our primary analysis of the effect of the intervention on smoking abstinence, we will use binary logistic regression. We will complete unadjusted and adjusted models testing for site-by-treatment interactions and assessing the clinically important predictors of level of nicotine dependence and education.

For our analysis of intervention effect on clinical events, we will use competing-risk regression [33]. Competing-risk regression compares the cumulative incidence of an event (i.e., hospitalization, ED visit) between two groups over a period of time in the presence of a competing risk (i.e., death). The primary independent variable in our models will be group (QCI vs. UC group).

We will also assess the effect of quitting on clinical outcomes, using smoking status as the independent variable in our models. An alpha level of 0.05 and two-tails will be used for all tests of significance and interval estimates will be based on 95% confidence intervals. We will use intention-to-treat principles and the Russell standard (the convention in cessation trials), removing patients who have deceased or become untraceable (e.g., number no longer in service, moved with no forwarding address or number) and assuming that those lost to follow-up (e.g., those who refuse contact) are smoking [34]. We will conduct per-protocol analyses using “engaged” participants (all who used their Quit Card and/or partook in ≥1 behavioral support contact). Analyses will be carried out using statistical software packages SAS (Cary, NC) and SPSS.

#### Interim analyses {21b}

Analyses will be completed at the primary endpoint (26 weeks), and at 52 weeks, and 104 weeks.

Each member of the research team, as well as the Data Safety and Monitoring Board (DSMB), will have access to interim results. As the co-investigators will remain blinded, group allocation will not be revealed during interim analyses.

#### Methods for additional analyses (e.g., subgroup analyses) {20b}

Several exploratory subgroup analyses are being planned a priori. Data will be stratified to estimate the effect of the intervention by age (quartiles), sex (male vs. female), gender (self-identified man vs. self-identified woman vs. other), socio-economic status (education level), nicotine dependency (high vs. low), treatment goal (quit abruptly vs. reduce to quit vs. reduce only vs. no goal), cannabis-use (regular cannabis smoker vs. non-cannabis smoker), and diagnosis (e.g., smoking-related vs. non-smoking related illness).

#### Methods in analysis to handle protocol non-adherence and any statistical methods to handle missing data {20c}

As described above, for the main outcome of smoking abstinence, with the exception of removing participants who have died, we will assume that those lost to follow-up (e.g., those who refuse contact) are smoking. For all other variables, under the assumption that missing data are missing at random (MAR), a missing variable imputation approach will be used.

#### Plans to give access to the full protocol, participant level-data, and statistical code {31c}

Access to the full study protocol, participant-level dataset, and statistical code used for data analysis will be available on request.

### Oversight and monitoring

#### Composition of the coordinating center and trial steering committee {5d}

UOHI’s Division of Prevention and Rehabilitation’s research manager will oversee issues related to research ethics submissions, human resource management, and issues that require knowledge of study allocation. The RC and assistants will report directly to the research manager. The RC will be responsible for recruitment, baseline data collection, data entry, and once recruitment is complete the RC will assist with follow-up surveys and CO monitor tests. The assistants will conduct the follow-up surveys, CO monitor tests, and data entry.

The principal investigator (KM) will be overseeing the study conduct and implementation and have ultimate responsibility regarding the trial. Co-investigator RR will assist with study design, analysis, and knowledge translation. Co-investigator AP will be one of three qualified investigators and will advise on medical issues, provide medical direction to the study counselor, and assist with knowledge translation. AK and VT, staff emergency medicine physicians at TOH, will be the two additional qualified investigators as well as site leads at both participating hospital campuses (The Ottawa Hospital Civic Campus and The Ottawa Hospital General Campus). Co-investigator GW will oversee activities of the Cardiovascular Research Methods Centre (CRMC), including trial design, randomization, and analysis. SH will assist with design, analysis, and data linkage activities. MK, the manager of the Ottawa Heart Institute’s Smoking Cessation Program, will provide direct support to the study counselor. KW, a PhD trainee, will contribute to Research Ethics Board (REB) submission, data analysis, and knowledge translation activities. The trial steering committee will be comprised of the principal investigator, co-investigators, and collaborators and will convene quarterly. In addition, the committee will oversee study design and conduct issues and publication of the results.

#### Composition of the data monitoring committee, its role, and reporting structure {21a}

A data safety and monitoring board (DSMB) will be established to review safety reports on a regular basis (the schedule of reports will be determined by the DSMB). The DSMB will be separate from the investigators, steering committee, and sponsoring research institute and will consist of at least 3 members with expertise in tobacco treatment, emergency medicine, and epidemiology or biostatistics.

#### Adverse event reporting and harms {22}

Adverse events and serious adverse events will be queried and documented using adverse event case report forms. All documented adverse events will be reviewed by the study PI and, if necessary, one of the three study qualified investigators. The qualified investigator would make the determination of whether or not the event was deemed related to the study. Research ethics would be notified. The DSMB will review all reported adverse events.

#### Frequency and plans for auditing trial conduct {23}

The DSMB will be responsible for auditing trial conduct annually.

#### Plans for communicating important protocol amendments to relevant parties (e.g., trial participants, ethical committees) {25}

Any modifications to the protocol which may impact study conduct, potential benefit of the patient, or may affect patient safety, including changes of study objectives, study design, patient population, sample sizes, study procedures, or significant administrative aspects will require a formal amendment to the protocol. Such changes will be agreed upon by the investigative team and approved by the REB prior to implementation and notification of the health authorities in accordance with provincial and federal regulations.

Administrative changes of the protocol are minor corrections and/or clarifications that have no effect on the way the study is to be conducted. These administrative changes will be agreed upon by the investigating team and will be documented in a memorandum. The REB may be notified of administrative changes at the discretion of the investigative team.

### Dissemination plans {31a}

The results of this trial will be released to clinicians, researchers, patients, and funding bodies. Assessment of health and healthcare utilization outcomes such as cost-effectiveness ratios and frequency of ED visits will be of particular interest to administrators and funders.

Knowledge will be shared using conventional academic approaches including high-impact peer-reviewed publications, through the OMSC’s annual conference, and via social media. In particular, the findings will be of interest to the >100 OMSC hospitals across Canada that are seeking strategies to assist smokers in the ED and other high-volume areas.

## Discussion

### COVID-19 amendments

Given challenges preventing on-site recruitment of participants directly in the ED during the COVID-19 pandemic, an amendment is being prepared to this protocol to implement virtual processes. It is being proposed that, each day, the RC will generate a report of all patients having been discharged from the ED within the last 24 h from the hospital electronic medical record (EMR) system. All patients who declined consent to be contacted for research purposes and those who have non-smoking status clearly identified on their record will be excluded. All others will be contacted by telephone, strictly in chronological order of ED arrival time, to assess smoking status, introduce the study, screen for eligibility, answer questions participants may have regarding the study and/or their participation, and obtain and document informed consent. The baseline questionnaire will be completed over the telephone, and the intervention will be randomly allocated in the same manner as previously planned, with the RC selecting the next available kit in the corresponding CTAS box. The RC will mail each participant their kit along with a copy of their informed consent form and will register the unique card number online into the Quit Card portal. For QCI group participants only, registration of the card number will activate their Quit Card and will lead to an electronic Quit Card being emailed to the study counselor. The counselor will then email the e-card to the participant and initiate the behavioral support.

Follow-up surveys will also be completed by telephone, and only those participants who self-report smoking abstinence at 26 weeks will be asked to complete a CO test to limit potential exposures.

#### CO monitoring

Extra precautions will be taken when performing exhaled CO tests with study participants. Medical-grade face masks, gloves, and a face shield shall always be worn by study staff to protect both themselves and participants. The CO monitor will only be handled by study staff wearing gloves, and the study team member performing the CO test will hold the device for the participant when they are blowing into the device (the device will never be handled by participants). The study team member performing the CO test will thoroughly wash their hands with soap and water immediately before and after performing the CO test, and the device will be wiped down with non-alcoholic antibacterial or viral wipes after each breath test. The device being used for this study is the Covita Micro+ Smokerlyzer. This device uses single-use mouthpieces containing a one-way valve and infection control filter that has been proven to remove and trap 99% of airborne bacteria and 96.5% of airborne viruses. The device’s single-use mouthpieces have been tested to filter viruses as small as 23 nanometres in diameter; therefore, theoretically, the mouthpiece should be effective at filtering out the COVID-19 virus particle which has been measured to have an approximate diameter of 125 nm, although testing of the single-use mouthpieces against COVID-19 has yet to be done. Additional information on this specific device’s safety features and the manufacturer’s suggestions specific to COVID-19 can be obtained by visiting the URL https://www.bedfont.com/coronavirus.

### Trial status

The protocol version is 4, dated Feb 24, 2020. Recruitment is expected to begin in February 2021.

## Abbreviations

ED: Emergency department
CIHR: Canadian Institute of Health Research
RCT: Randomized controlled trial
QCI: “Quit Card” intervention
UC: Usual care
CTAS: Canadian Triage and Acuity Scale
HR-QoL: Health-related quality of life
QSP: University of Ottawa Heart Institute’s Quit Smoking Program
NRT: Nicotine replacement therapy
NATS: Nicotine addiction treatment specialist
UOHI: University of Ottawa Heart Institute
OHRI: Ottawa Hospital Research Institute
OHIRC: Ottawa Heart Institute Research Corporation
CRMC: Cardiovascular Research Methods Centre
MI: Motivational interviewing
OR: Odds ratio
CI: Confidence interval
RR: Relative risk
SES: Socioeconomic status
ICES: Institute for Clinical Evaluative Sciences
CHEO: Children’s Hospital of Eastern Ontario
RC: Research coordinator
TOH: The Ottawa Hospital
ICF: Informed Consent Form
OMSC: Ottawa Model for Smoking Cessation
CO: Carbon monoxide
EMR: Electronic medical record
PI: Principal investigator
Co-I: Co-investigator
CIHI: Canadian Institutes of Health Information
IRSS: Institute for Health Services Research
REB: Research Ethics Board
DSMB: Data Safety and Monitoring Board
OHIP: Ontario Health Insurance Plan
